# Construction of a risk screening and visualization system for pulmonary nodule in physical examination population based on feature self-recognition machine learning model

**DOI:** 10.3389/fmed.2024.1424750

**Published:** 2025-03-04

**Authors:** Fang Tian, Yongchun Lin, Liangjiao Wang, Fei Fang, Kaiwen Hou

**Affiliations:** ^1^Department of Outpatient, Western Theater Command General Hospital of PLA, Chengdu, Sichuan, China; ^2^Department of Emergency, Tibet Command General Hospital of PLA, Lhasa, China

**Keywords:** machine learning, pulmonary nodules, risk screening, visualization system, algorithm

## Abstract

**Objective:**

To assess the effectiveness of a feature self-recognition machine learning model in screening for pulmonary nodule risk in a physical examination population and to evaluate the constructed visualization system.

**Methods:**

We analyzed data from 4,861 individuals who underwent chest CT exams during their physical examinations at the Western Theater General Hospital of the People’s Liberation Army from January 2023 to November 2023. Among them, 1,168 had positive CT reports for pulmonary nodules, while 3,693 had negative findings. We developed a machine learning model using the XGBoost algorithm and employed an improved sooty tern optimization algorithm (ISTOA) for feature selection. The significance of the selected features was evaluated through univariate analysis and multivariable logistic stepwise regression analysis. A visualization system was created to estimate the risk of developing pulmonary nodules.

**Results:**

Multivariable analysis identified older age, smoking or passive smoking, high psychological stress within the past year, occupational exposure (e.g., air pollution at the workplace), presence of chronic lung diseases, and elevated carcinoembryonic antigen levels as significant risk factors for pulmonary nodules. The feature self-recognition machine learning model further highlighted age, smoking or passive smoking, high psychological stress, occupational exposure, chronic lung diseases, family history of lung cancer, decreased albumin levels, and elevated carcinoembryonic antigen as key predictors for early pulmonary nodule risk, demonstrating superior performance.

**Conclusion:**

The feature self-recognition machine learning model effectively aids in the early prediction and clinical identification of pulmonary nodule risk, facilitating timely intervention and improving patient prognosis.

## 1 Introduction

The widespread implementation of lung cancer screening programs has markedly increased the detection rates of pulmonary nodules. These nodules, characterized as focal, round-shaped, solid or subsolid lung opacities not exceeding 3 cm in diameter on imaging, can evolve into malignant tumors if not diagnosed and managed promptly. This progression significantly deteriorates the quality of life for affected individuals (1, 2). Lung cancer remains the most prevalent and deadliest of all malignant tumors, with most patients presenting at advanced stages, resulting in low five-year survival rates and poor prognoses (3, 4). Consequently, the effective management of pulmonary nodules is crucial in the prevention and control of lung tumors.

The clinical manifestations of pulmonary nodules are non-specific, complicating the diagnostic process and increasing the likelihood of misdiagnosis. Traditionally, the assessment of these nodules for benign or malignant characteristics involves the analysis of chest CT images or the employment of invasive techniques such as surgery or biopsy to obtain a definitive lesion characterization (5, 6). However, recent advancements in artificial intelligence (AI) have facilitated the extraction of feature information and the development of predictive models. These innovations are proving instrumental in aiding physicians to diagnose suspicious pulmonary nodules non-invasively. Such technological progress not only enhances the potential for early disease detection and prognosis but also significantly improves the diagnostic accuracy of pulmonary nodules (7, 8). For instance, studies have demonstrated that deep learning models are capable of learning subtle image features from complex imaging data, features that are often elusive to traditional methods (2, 9). These advancements have not only improved the accuracy in distinguishing benign from malignant pulmonary nodules but have also shortened the diagnostic process, providing quicker decision support for patient treatment (10, 11). Furthermore, recent research has explored how improvements in algorithms and model structures can enhance the generalizability and interpretability of diagnostic systems, making their application in clinical practice more widespread and effective (12). These findings not only confirm the potential of artificial intelligence technology in non-invasive diagnostics but also highlight future research directions, specifically how to better integrate these advanced technologies into routine clinical diagnostic processes to improve early disease detection and treatment outcomes (13). The aim of this study is to analyze the value of pulmonary nodules risk screening in physical examination population and the effect of visualization system construction based on feature self-recognition machine learning mode.

## 2 Methods and materials

### 2.1 Study population

A total of 4,861 individuals who underwent chest CT examinations as part of their physical examinations at the Western Theater General Hospital of the People’s Liberation Army from January 2023 to November 2023 were included in this study, access to the study data began on January 1, 2024. Among them, 1,168 patients had positive CT reports for pulmonary nodules, while 3,693 patients had negative findings. Inclusion criteria were as follows: (1) Normal mental status, clear cognition, and able to cooperate with inquiries; (2) Complete clinical data. Exclusion criteria were as follows: (1) Presence of severe diseases such as cardiovascular, liver, or kidney disorders; (2) History of previous tumors; (3) Pregnant or breastfeeding women.

This study was conducted retrospectively, informed consent was waived, and this study was approved by the Ethics Committee of the Western Theater General Hospital of the People’s Liberation Army (Approval No.: 2022ky105-3). All data were anonymized.

### 2.2 Data collection

General information and laboratory test results of the study participants were obtained from the Health Management System of the Western Theater General Hospital of the People’s Liberation Army, comprising a total of 33 features. The general information included gender, age, smoking history, alcohol consumption history, place of residence, education level, chronic lung diseases, family history of lung cancer, regular exercise, body mass index, presence of high psychological stress within the past year, and presence of depressive symptoms within the past year. The laboratory test results included carcinoembryonic antigen, thyroid-stimulating hormone, white blood cell count, lymphocyte count, platelet count, hemoglobin, eosinophil count, basophil count, albumin, globulin, albumin-globulin ratio, alanine aminotransferase, aspartate aminotransferase, indirect bilirubin, high-density lipoprotein, low-density lipoprotein, triglycerides, fasting blood glucose, creatinine, blood urea nitrogen, and uric acid.

### 2.3 Feature self-recognition machine learning model

**Figure 1:**
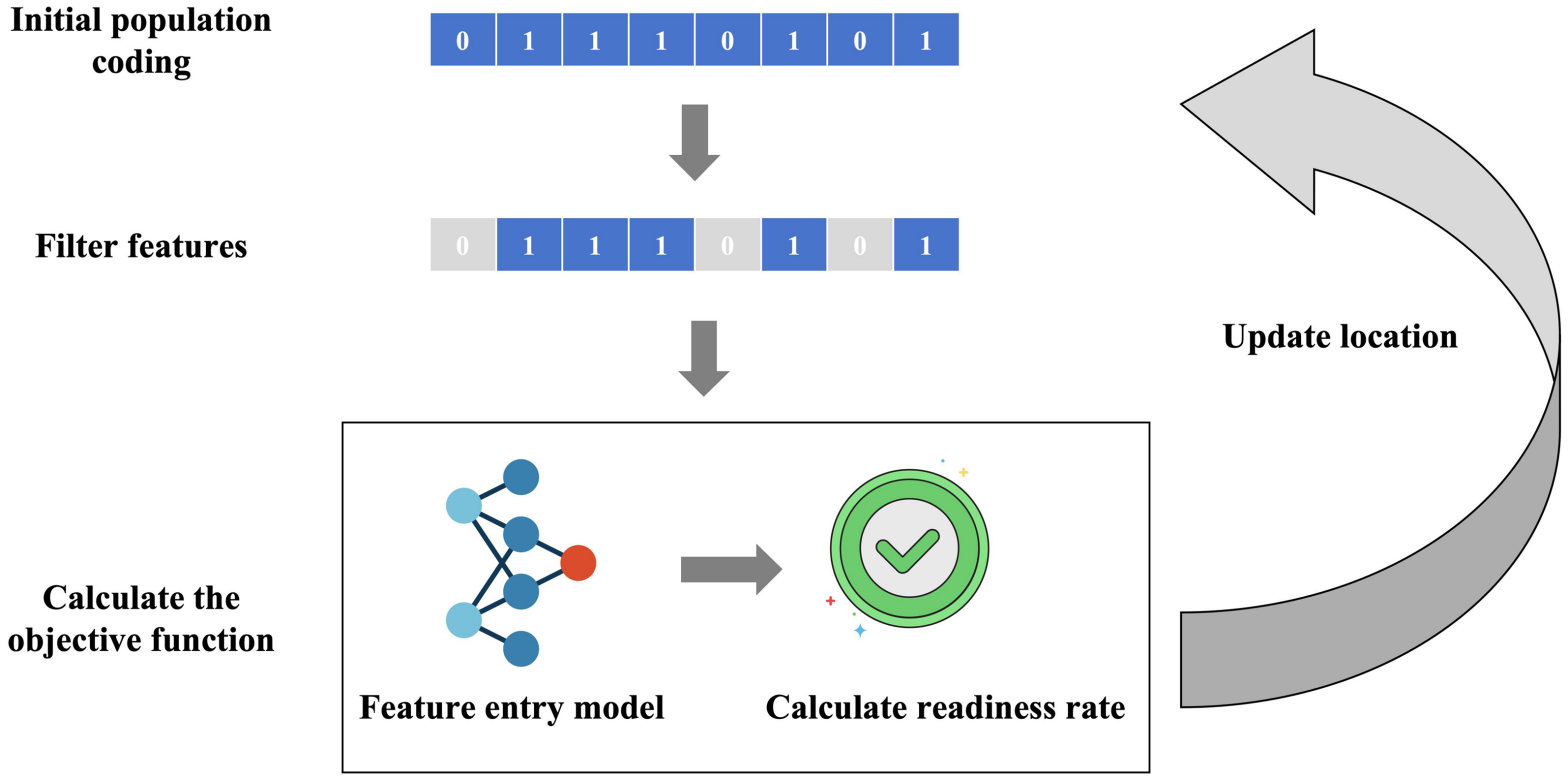
Schematic diagram of the FSRML model. Firstly, swarm intelligence algorithms initialize the generation of chaotic population random sequences. Each chaotic sequence can be filtered using binary encoding (0 represents feature deletion, 1 represents feature retention). The filtered features are then fed into the machine learning model, and the predictive accuracy is calculated as the objective function. Through the iteration of the swarm intelligence algorithm’s population, the feature selection and model construction results are gradually optimized.

In this study, we proposed a feature self-recognition machine learning model (FSRML) that does not require preliminary feature selection before running. All 33 features studied were included in the model training. The FSRML utilizes the powerful global optimization capability of swarm intelligence algorithms to automatically perform feature selection. The schematic diagram of the model is shown in Figure 1.

Caption: Firstly, swarm intelligence algorithms initialize the generation of chaotic population random sequences. Each chaotic sequence can be filtered using binary encoding (0 represents feature deletion, 1 represents feature retention). The filtered features are then fed into the machine learning model, and the predictive accuracy is calculated as the objective function. Through the iteration of the swarm intelligence algorithm’s population, the feature selection and model construction results are gradually optimized.

To better guide the feature optimization task mentioned above, this study improves upon the sooty tern optimization algorithm (STOA) (14) by incorporating three enhancement strategies: Bernoulli chaotic mapping (15), Cauchy mutation perturbation (16), and longitudinal-lateral crossover mutation (17). These improvements lead to the development of a hybrid chaotic sooty tern optimization algorithm that combines longitudinal-lateral crossover and Cauchy mutation. It is referred to as the improved sooty tern optimization algorithm (ISTOA). The specific improvement strategies are as follows:

(1) Bernoulli chaotic mapping

Swarm intelligence optimization algorithms generally generate populations through randomization. However, when the population size is small, the populations generated by random arrays may lack sufficient ergodicity, potentially causing the optimization results to fall into local optima. Chaotic sequences, characterized by strong ergodicity, unpredictability, and sensitivity to initial values, are better suited for the task of initializing populations. Bernoulli mapping is a typical example of chaotic mapping, and its expression is as follows:


$$x_{n+1}=\{\begin{array}{l} \frac{x_{n}}{1-\lambda }\;0<x_{n}<1-\lambda \\ \frac{x_{n}-\left(1-\lambda \right)}{\lambda }\;1-\lambda <x_{n}<1 \end{array}$$

In the above expression, we set the value to 0.4. First, a random number *x_0_* between 0 and 1 is generated. Then, the chaotic sequence is produced according to the aforementioned formula.

(2) Cauchy mutation disturbance

Based on the original STOA, we set a certain probability to perform a position update using Cauchy mutation disturbance. This enhances the algorithm’s ability to escape local optima. The formula for the Cauchy probability density function is as follows:


$$f\left(x;x_{0},\lambda \right)=\frac{1}{\pi \lambda \left[1+{\left(\frac{x-x_{0}}{\lambda }\right)}^{2}\right]}=\frac{1}{\pi }\left[\frac{\lambda }{{\left(x-x_{0}\right)}^{2}+\lambda^{2}}\right]$$

Incorporating this into the STOA position update formula, we have:


$$X_{\mathrm{newbest}}=X_{\mathrm{best}}+X_{\mathrm{best}}\times \mathrm{Cauchy}\left(0,1\right)$$

where Cauchy() represents the Cauchy probability density function, *X_newbest_* is the position after mutation, and *X_best_* is the best position before mutation.

### 2.4 Software system development

A visualization prediction system was built based on the constructed predictive model. This system was developed using MATLAB R2022a and designed using the APP Designer functionality, resulting in an initial *.mlapp file. Subsequently, the *.mlapp file was compiled into an executable *.exe file that can run independently without the need for the MATLAB environment. As long as the computer has MATLAB Runtime installed, the software can be run, effectively reducing the software’s runtime environment requirements and improving its portability.

### 2.5 Statistical analysis

The predictive model construction was performed using MATLAB 2022a, and data analysis was conducted using SPSS 26.0 software. A significance level of *p* < 0.05 was used to indicate statistically significant differences. Count data were presented as [n (%)] and compared using the chi-square test, while normally distributed continuous data were expressed as (mean ± standard deviation) and compared using the t-test.

## 3 Results

### 3.1 Performance testing of ISTOA optimization

**Figure 2:**
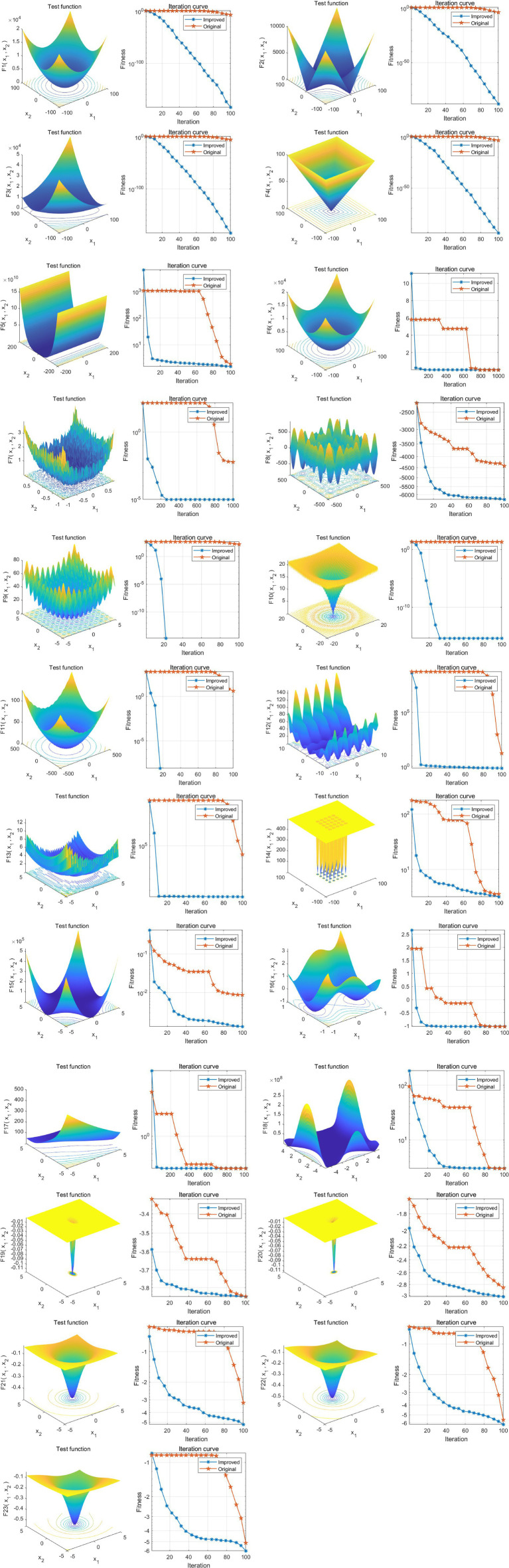
Performance analysis of ISTOA on 23 standard benchmark functions. The 3D surface plots in the figure depict the two-dimensional search space for each benchmark function. The convergence curves illustrate the convergence trends of the first solution dimension for each benchmark function, with a comparison between STOA (blue curve) and the improved ISTOA (red curve).

To comprehensively evaluate the performance and efficiency of various algorithms, this study utilized 23 standard benchmark functions to assess the performance of each algorithm, the specific 23 functions are shown in Supplementary File 1. The results demonstrated that ISTOA exhibited significantly faster convergence speed and superior global optimization capability compared to other algorithms. Please refer to Figure 2a for detailed findings. Thus, the ISTOA algorithm showed clear advantages in optimization, making it suitable as the guiding algorithm for feature automatic selection in this study.

### 3.2 Lung nodule prediction model construction

#### 3.2.1 Model construction overview

Randomly selecting 80% of the dataset as the training set (3,889 cases), we chose several base models including logistic regression (LR), decision tree (DT), k-nearest neighbors algorithm (KNN), backpropagation neural network (BP), support vector machine (SVM), random forest (RF), and XGBoost. All these models were integrated with ISTOA for automatic feature selection, thus constructing the FSRML model. Five-fold cross-validation was performed for all models. Comparing the validation results of the five-fold cross-validation, it was evident that XGBoost exhibited significant advantages (Table 1; Figures 2b,c).

**Table 1:** Cross-validation results of FSRML model training (validation set).

Basic model	PRE	SEN	SPE	ACC	F1	ROC-AUC	PR-AUC
LR	0.4016	0.0525	0.9753	0.7537	0.0928	0.6866	0.3681
DT	0.5602	0.4979	0.8765	0.7855	0.5272	0.7801	0.5379
KNN	0.5519	0.6660	0.8291	0.7899	0.6036	0.8480	0.6187
BP	0.6779	0.7548	0.8866	0.8550	0.7143	0.8852	0.7247
SVM	0.6131	0.4497	0.9103	0.7997	0.5188	0.8118	0.5504
RF	0.8893	0.6970	0.9726	0.9064	0.7815	0.9323	0.8726
XGBoost	0.9410	0.6831	0.9865	0.9136	0.7916	0.9496	0.9028

#### 3.2.2 Machine learning model performance testing

**Figure 3:**
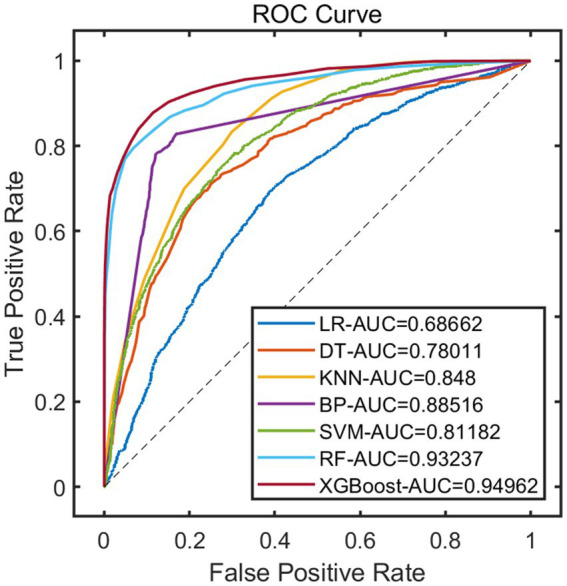
ROC curve of validation set.

**Figure 4:**
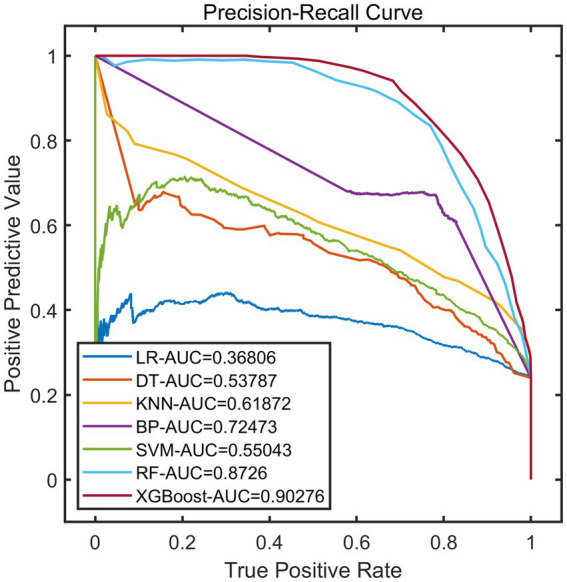
PR curves of validation set.

After constructing the models, the remaining 20% of the samples (972 cases) were selected as the test set to evaluate the predictive performance of each model on external data. The results showed that using XGBoost as the base model for FSRML yielded significant improvements in predictive performance (Table 2; Figures 3, 4).

**Table 2:** FSRML model performance comparison results (test set).

Basic model	PRE	SEN	SPE	ACC	F1	ROC-AUC	PR-AUC
LR	0.3030	0.0427	0.9688	0.7459	0.0749	0.6706	0.3614
DT	0.5490	0.5983	0.8442	0.7850	0.5726	0.7973	0.5231
KNN	0.6547	0.6239	0.8957	0.8302	0.6389	0.8647	0.6460
BP	0.7017	0.7137	0.9038	0.8580	0.7076	0.8969	0.6757
SVM	0.6575	0.4103	0.9322	0.8066	0.5053	0.8060	0.5710
RF	0.9483	0.7051	0.9878	0.9198	0.8088	0.9407	0.8958
XGBoost	0.9148	0.6880	0.9797	0.9095	0.7854	0.9522	0.9077

#### 3.2.3 Compared with other automatic machine learning methods

**Figure 5:**
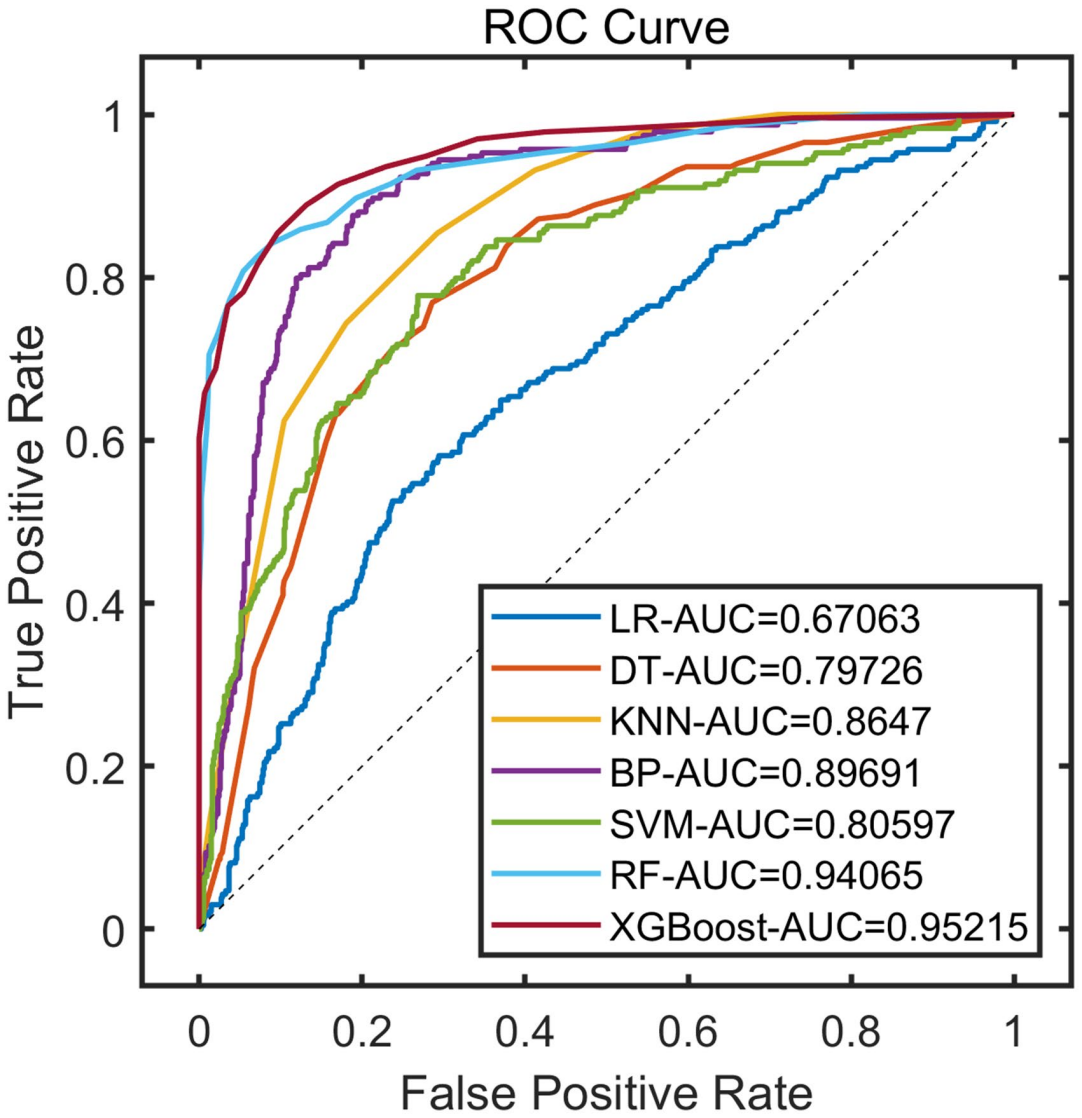
ROC curve of test set.

**Figure 6:**
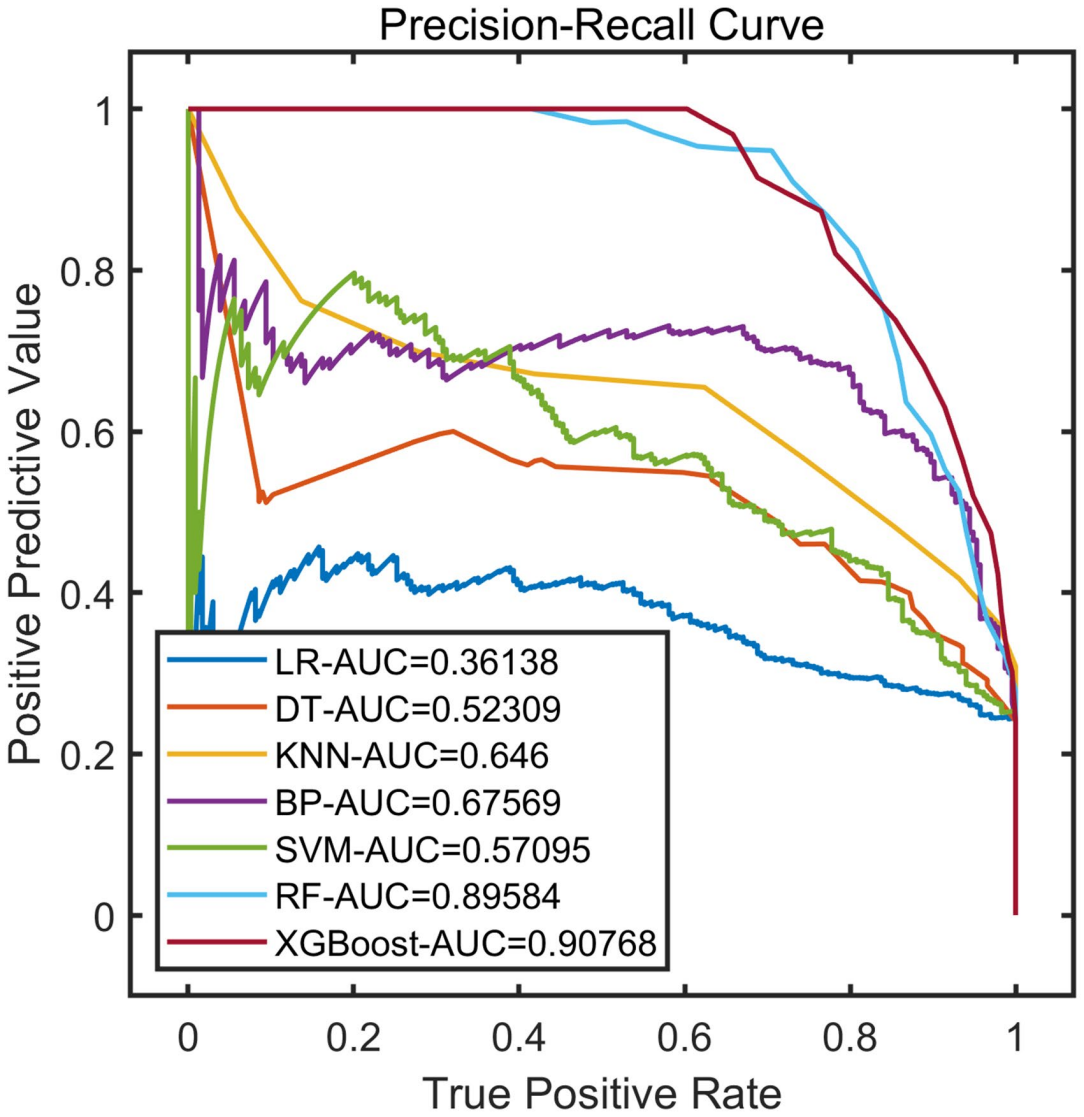
PR curve of test set.

The FSRML we developed was compared with other AutoML models, and the results showed that the FSRML model constructed in this study had the best prediction performance on the test set (Table 3; Figures 5, 6).

**Table 3:** Comparison of FSRML and other AutoML prediction performance.

Model	PRE	SEN	SPE	ACC	F1	ROC-AUC	PR-AUC
TPE-GP	0.7778	0.0598	0.9946	0.7695	0.1111	0.7112	0.4535
TPOT	0.9412	0.2735	0.9946	0.8210	0.4238	0.9242	0.8220
AutoSklearn	0.3529	0.0256	0.9851	0.7541	0.0478	0.6865	0.3637
AutoGluon	0.6329	0.6410	0.8821	0.8241	0.6369	0.8431	0.6755
FSRML	0.9148	0.6880	0.9797	0.9095	0.7854	0.9522	0.9077

### 3.3 Feature validation through model-automated selection

The features selected automatically by the FSRML model include age, smoking or frequent passive smoking, significant psychological stress in the past year, occupational exposure (presence of air pollution in the work environment), presence of chronic lung disease, family history of lung cancer, elevated levels of albumin, and elevated levels of carcinoembryonic antigen. The value of these selected features was assessed through univariate analysis and multivariate logistic stepwise regression analysis.

#### 3.3.1 Univariate analysis for feature selection

The results of the univariate analysis showed that in patients with positive lung nodules, the proportions of age, smoking or frequent passive smoking, significant psychological stress in the past year, occupational exposure (presence of air pollution in the work environment), presence of chronic lung disease, family history of lung cancer, and elevated levels of carcinoembryonic antigen were higher compared to patients with negative lung nodules. Additionally, the level of albumin was lower in patients with positive lung nodules. These differences were statistically significant (*p* < 0.05) (Table 4).

**Table 4:** Results of univariate analysis of model selection feature.

Feature	Negative (*n* = 3,693)	Positive (*n* = 1,168)	*t*/*χ*^2^ value	*p-*value
Age(year)	33.87 ± 8.43	43.32 ± 9.16	32.691	<0.001
Smoking or frequent passive smoking			320.600	<0.001
Yes	1,469(39.78)	815(69.78)		
No	2,224(60.22)	353(30.22)		
Significant psychological stress in the past year			29.163	<0.001
Yes	775(20.99)	327(28.66)		
No	2,918(79.01)	814(71.34)		
Occupational exposure (presence of air pollution in the work environment)			209.891	<0.001
Yes	443(12.0)	350(29.97)		
No	3,250(88.0)	818(70.03)		
Presence of chronic lung disease			41.936	<0.001
Yes	187(5.06)	121(10.36)		
No	3,506(94.94)	1,047(89.64)		
Family history of lung cancer			136.618	<0.001
Yes	517(14.0)	338(28.94)		
No	3,176(86.0)	830(71.06)		
Albumin (g/L)	49.14 ± 11.23	44.92 ± 9.33	11.635	<0.001
Elevated levels of carcinoembryonic antigen			40.807	<0.001
Yes	61(1.65)	58(4.97)		
No	3,632(98.35)	1,110(95.03)		

#### 3.3.2 Multivariate analysis for feature selection

The results of the multivariate analysis showed that advanced age, smoking or frequent passive smoking, significant psychological stress in the past year, occupational exposure (presence of air pollution in the work environment), presence of chronic lung disease, and elevated levels of carcinoembryonic antigen were identified as risk factors for predicting the occurrence of lung nodules (Table 5).

**Table 5:** Results of multivariate logistic regression stepwise regression analysis.

Feature	β	SE	Wald	*P*	EXP(B)	95% CI for EXP(B)	
						Lower	Upper
Age (year)	1.536	0.636	5.833	0.019	4.646	3.399	5.893
Smoking or frequent passive smoking	1.231	0.311	15.667	<0.001	3.425	2.815	4.034
Significant psychological stress in the past year	0.515	0.134	14.771	<0.001	1.674	1.411	1.936
Presence of chronic lung disease	0.742	0.237	9.802	0.003	2.100	1.636	2.565
Elevated levels of carcinoembryonic antigen	1.011	0.352	8.249	0.006	2.748	2.058	3.438
Occupational exposure (presence of air pollution in the work environment)	1.067	0.491	4.722	0.034	2.907	1.944	3.869

### 3.4 Development of visualization system

In clinical practice, the changes in various features related to lung nodules can be complex and difficult to visually interpret, making it challenging to determine whether a patient is at risk of developing lung nodules. Existing artificial intelligence methods also face the challenge of high implementation barriers, requiring clinicians to possess advanced coding skills and extensive literature review, which hinders widespread adoption in hospitals. To address this issue, this study innovatively developed a practical visualization system called “A Risk Prediction System for Pulmonary Nodules in Physical Examination Population.” This system offers intuitive, convenient, and practical advantages.

**Figure 7:**
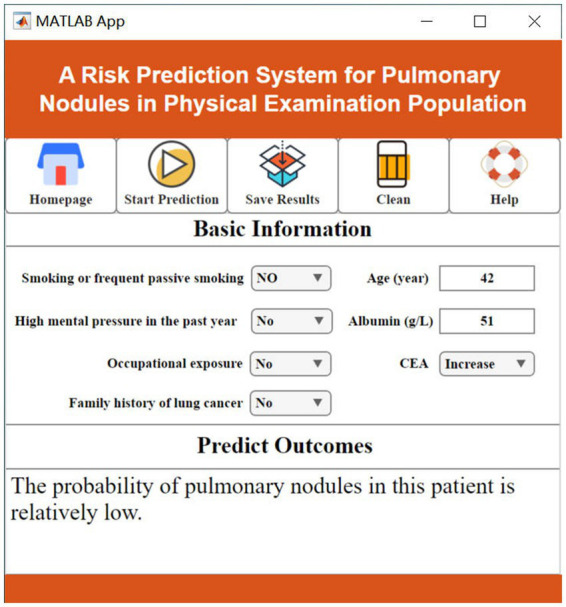
Low risk of pulmonary nodules.

**Figure 8:**
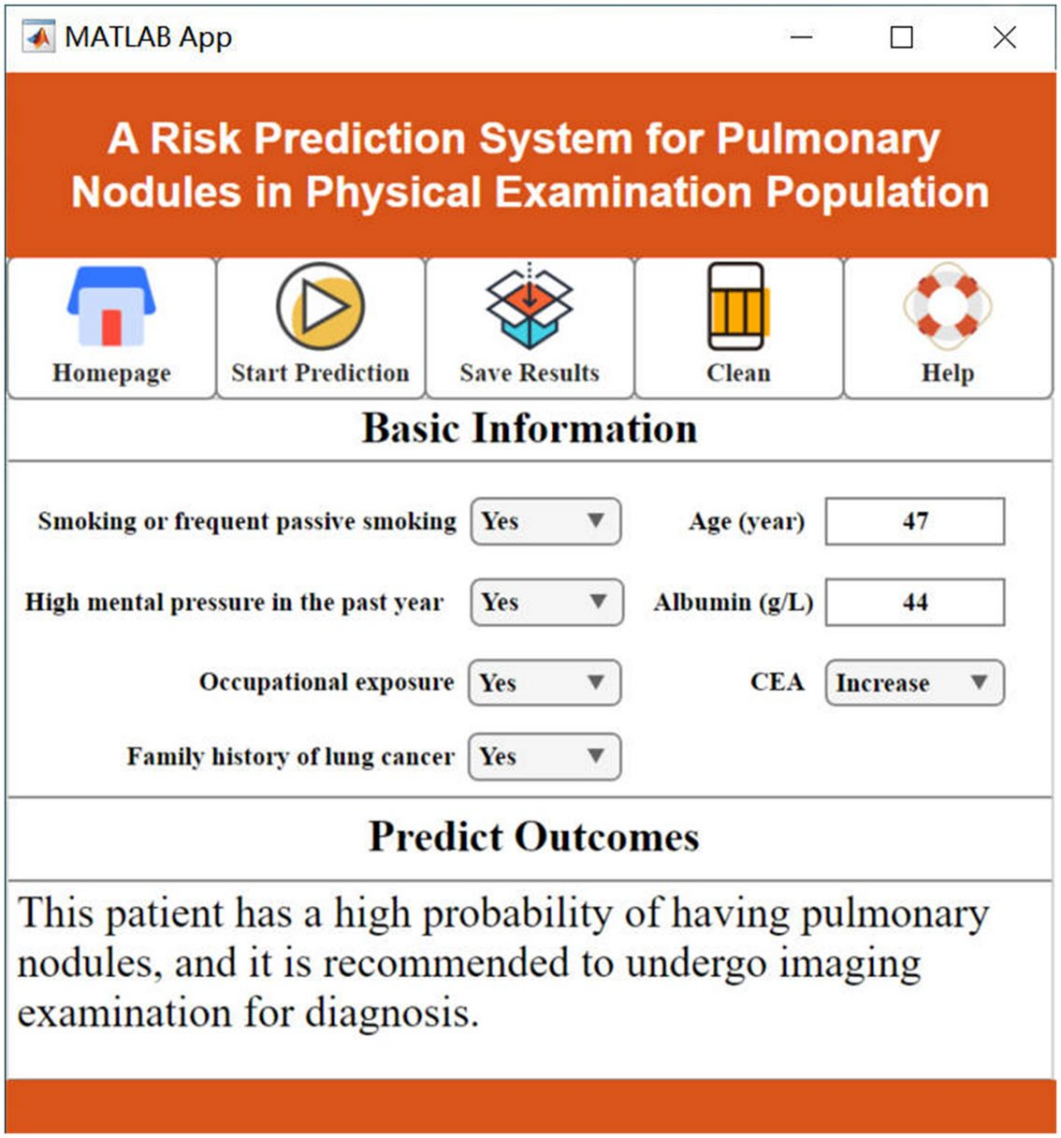
High risk of pulmonary nodules.

Users can input patients’ basic information in the “Basic Information Input” section and then click the “Prediction” button. The predicted results will be displayed in the “Prediction Result Output” section, providing users with easy access to the prediction outcomes (Figures 7, 8).

## 4 Discussion

In recent years, with changes in people’s lifestyles and the influence of environmental factors, the incidence of lung nodules, as one of the early signs of lung cancer, has been increasing year by year (18). Currently, the diagnostic techniques for lung nodules mainly include CT scanning, needle biopsy, or pathological examination after surgery. CT scans rely on comprehensive analysis by physicians of lesion location, size, density, shape, and other information to make a qualitative diagnosis. However, different pathological subtypes of lung nodules often exhibit similar imaging features, and the diagnosis of the same lesion may be influenced by subjective differences among different diagnosticians, making it difficult to accurately diagnose early-stage lung cancer (19, 20). Pathological examination is considered the gold standard for diagnosing lesions, but it does not provide a comprehensive assessment of the entire lesion. Therefore, different pathological results can also occur depending on the site of sample collection (21). Positron Emission Tomography/Computed Tomography (PET/CT)，the significant role in the evaluation and management of pulmonary nodules. PET/CT is instrumental in distinguishing between benign and malignant nodules, enhancing the diagnostic accuracy beyond what is achievable with CT alone. This imaging modality integrates metabolic and anatomic information, providing a more comprehensive assessment of nodule activity. Studies have shown that PET/CT can significantly improve the sensitivity and specificity of lung cancer detection, especially in nodules that are indeterminate in size and appearance (22). However, the high cost of PET/CT makes it difficult to promote it in clinical practice.

Machine learning is an interdisciplinary field that combines statistics, various domains of knowledge, and computer technology to process large volumes of data. It is a subfield of artificial intelligence. By utilizing machine learning algorithms, researchers can extract the necessary feature variables from massive datasets, thereby enhancing learning efficiency (23, 24). Machine learning has been widely applied in the medical field. In this study, a machine learning model based on XGBoost was developed for feature recognition. This model automates the preliminary work of machine learning, including data preparation, encoding, feature selection/extraction, and engineering environment. During the model generation process, it involves algorithm selection, optimization, iteration, and validation (25, 26). Additionally, this study utilized the ISTOA for optimizing the performance of machine learning. This algorithm builds upon the decision tree algorithm and continuously improves precision through accumulation (27). An essential aspect of implementing data-driven models in medicine is ensuring the feasibility and integration of these processes within healthcare service providers. The successful deployment of our feature self-recognition machine learning model for pulmonary nodule risk screening hinges not only on its predictive accuracy but also on its practical application in clinical settings. According to recent studies, it is crucial to consider factors such as interoperability with existing healthcare systems, ease of use for clinical staff, and the ability to handle large-scale data efficiently. Our model has been designed with these considerations in mind, featuring an intuitive visualization system that can seamlessly integrate with electronic health records (EHR) and other hospital information systems (HIS). Additionally, the model’s reliance on routinely collected clinical data ensures that its implementation does not require significant changes to current workflows, thereby facilitating its adoption in real-world healthcare environments. Future work will focus on pilot testing the system in various healthcare settings to further validate its feasibility and gather feedback for continuous improvement.

In this study, both univariate analysis and multivariate logistic regression models were used to identify six influencing factors: advanced age, smoking or frequent passive smoking, significant psychological stress in the past year, occupational exposure (presence of air pollution in the work environment), presence of chronic lung disease, and elevated levels of carcinoembryonic antigen. On the other hand, the feature recognition machine learning model identified eight features, including age, smoking or frequent passive smoking, significant psychological stress in the past year, occupational exposure (presence of air pollution in the work environment), presence of chronic lung disease, family history of lung cancer, decreased levels of albumin, and elevated levels of carcinoembryonic antigen. These features can be used for early diagnosis and prediction of the risk of developing lung nodules. This is because in the regression models, there is a high degree of linear correlation among the independent variables, which leads to inaccurate, unstable, and even unreliable estimates of the regression coefficients. This affects the predictive ability of the models and indicates that machine learning outperforms traditional multivariate analysis.

An analysis of the aforementioned risk factors reveals that both men and women have an increased incidence of lung nodules with age. This is because as the body ages, the immune system weakens, cell self-repair capabilities decline, and various carcinogenic factors stimulate the development of multiple diseases, promoting tumor growth (28, 29). Smoking intensity and duration are positively correlated with the incidence of lung nodules. This is due to the presence of dozens of carcinogens in tobacco, which can cause genetic mutations and promote chronic tumor growth. Passive smokers unknowingly inhale smoke, leading to lung function impairment (30, 31). Smoking also causes constriction of small blood vessels in the lungs and thickening of vessel walls, resulting in elevated levels of carcinoembryonic antigen (32). Air pollution and smoking have a synergistic effect on the occurrence and development of lung cancer, continuously increasing the incidence of lung nodules. Previous studies have shown that exposure to kitchen fumes and occupational exposure increase the risk of developing lung nodules. This is because kitchen fumes mainly contain carcinogens such as benzopyrene, volatile nitrosamines, and heterocyclic amine compounds, which exert cytotoxic effects on lung tissue and damage the respiratory system. Occupational exposure to substances like aluminum, arsenic, asbestos, coke, and coal gas has carcinogenic effects on the lungs (33, 34). Most lung nodules are caused by lung inflammation, and underlying lung diseases such as pneumonia, emphysema, chronic bronchitis, chronic obstructive pulmonary disease (COPD), and asthma are all inflammatory conditions that can recur and increase the incidence of lung nodules (35, 36). A positive family history of lung cancer and a history of lung disease are positively associated with the development of lung nodules, increasing the risk of their occurrence (37). Reasons for low albumin levels include inadequate intake, excessive consumption, excessive elimination, and insufficient synthesis. In patients with lung nodules, low albumin levels can be caused by malnutrition, impaired liver function, tumor metastasis, digestive tract tumors, liver tumors, and other factors (38, 39).

Compared to traditional statistical models such as logistic regression, the feature recognition machine learning model improves model accuracy. Additionally, the use of the ISTOA significantly reduces the barrier to entry for artificial intelligence technology. Healthcare professionals can utilize this tool to screen individuals undergoing medical examinations for their risk of developing lung nodules and implement targeted intervention measures. For example, they can promote a balanced diet, encourage physical exercise, foster healthy lifestyle habits, and establish regulations to restrict smoking (40, 41).

## 5 Conclusion

The application of feature recognition machine learning models can help clinicians identify characteristics of lung nodule patients, thereby enabling early prediction of disease occurrence, assisting in the development of treatment plans, and improving prognosis. However, this study also has certain limitations. Firstly, it is a retrospective and single-center study, which may introduce selection bias and affect the accuracy of the research findings. To further validate the results, more multicenter sample data is needed. Additionally, CT imaging plays a crucial role in the diagnosis of lung nodules in clinical practice. However, this study did not include CT imaging radiomics features, indicating the need for further analysis in future studies.

## Data Availability

The raw data supporting the conclusions of this article will be made available by the authors, without undue reservation.
